# The molecular mechanism of ovarian granulosa cell tumors

**DOI:** 10.1186/s13048-018-0384-1

**Published:** 2018-02-06

**Authors:** Jiaheng Li, Riqiang Bao, Shiwei Peng, Chunping Zhang

**Affiliations:** 1 Joint programme of Nanchang University and Queen Mary University of London, Nanchang, China; 2 Department of Gynecology and Obstetrics, Jiangxi Provincial People’s Hospital, Nanchang, China; 3 Department of Cell Biology, School of Medicine, Nanchang University, Nanchang, Jiangxi 330006 People’s Republic of China

**Keywords:** GCT, FOXL2, PI3K/AKT signaling, TGF-β signaling, Notch signaling

## Abstract

Over these years, more and more sex cord-stromal tumors have been reported. Granulosa cell tumor (GCT) is a rare tumor in ovaries, accounts for 2% to 5% of ovarian cancers. The main different feature of GCTs from other ovarian cancers is that GCTs can lead to abnormally secreted hormones (estrogen, inhibin and Müllerian inhibiting substance). The GCT is divided into two categories according to the age of patients, namely AGCT (adult granulosa cell tumor) and JGCT (Juvenile granulosa cell tumor). AGCT patients accounts for 95%. Although the pathogenesis is not clear, FOXL2 (Forkhead box L2) mutation was considered as the most critical factor in AGCT development. The current treatment is dominated by surgery. Target therapy remains in the adjuvant therapy stage, such as hormone therapy. During these years, other pathogenic factors were also explored, such as PI3K/AKT (phosphatidylinositol-3-kinase; serine/threonine kinase), TGF-β (Transforming growth factor beta) signaling pathway, Notch signaling pathway, GATA4 and VEGF (vascular endothelial growth factor). These factors and signaling pathway play important roles in GCT cell proliferation, apoptosis, or angiogenesis. The purpose of this review is to summarize the possible pathogenic factors and signaling pathways, which may shed lights on developing potential therapeutic targets for GCT.

## Background

Granulosa cell tumor (GCT) is the most common sex cord-stromal tumor that stem from granulosa cells. GCT accounts for 2% to 5% of all ovarian cancers and can be divided into two subtypes according to the differences of the age of patients, clinical and histopathologic features [1]. About 95% of GCT belong to the adult granulosa cell tumors (AGCTs), and others are juvenile granulosa cell tumors (JGCTs). JGCT only occurs in people who are younger than 30 years old with the features of hypoestrogenism and abnormal abdominal mass [2, 3]. Clinical features of AGCT include abnormal uterine bleeding in postmenopausal patients and menometrorrhagia in youngers. Some reports also indicated that patients were with stopping ovulating symptom [4]. The incidence of GCT is around 0.47 to 1.6 per 100,000. The main risk factors of GCT include nulliparity, fatness, oral contraceptives and family cancer history. From the cancer databases in Finland, Iceland, Norway and Sweden, the GCT onset showed scattered feature. There was no increasing trend over the 60 years [5]. Abnormal cell cycle is related to the occurrence and development of cancers. The recent studies provided powerful evidences that fork head box protein L2 (FOXL2), PI3K/AKT signaling pathway, TGF-β signaling pathway, Notch signaling pathway and etc. were involved in granulosa cell tumor through influencing cell proliferation and apoptosis [6–10].

In the development of GCT, a variety of cell signaling pathways, such as TGF-β, Notch and PI3K/AKT, are involved. In fact, these signal pathways are not isolated, but make up a complex network and contribute to the formation and development of GCT. FOXL2 is the most important mutant gene in GCT formation. Studies showed that FOXL2 is involved in the TGF-β pathway. For example, FOXL2 mutation has negative effect on SMAD3 (drosophila mothers against decapentaplegic protein) activation by interacting with BMPs, follistatin and activin A [11]. FOXO1/3 (forkhead box O1/3) also inhibited SMAD3 [12]. The interaction between Notch signaling and PI3K/AKT were also proved [13]. In the following sections, we will summarize the influence of different cell signaling network on GCT.

### FOXL2

Forkhead transcription factor 2 (FOXL2) is a transcription factor. The gene is 2.7 kb long and encodes 376 amino acids, which locates at human chromosome 3q23. The sequence of FOXL2 is highly conserved. It is mainly expressed in ovarian granulosa cell and pituitarium. FOXL2 is the first confirmed autosomal gene that maintains normal function of ovary, and it is also a marker of sexual selection and development. FOXL2 knockout mouse model showed sex reversal [14]. Further studies showed that FOXL2 regulated the ovarian granulosa cell proliferation, follicle development and ovarian hormones synthesis [14].

In 2009, a breakthrough of AGCT, using the whole-transcriptome paired-end RNA sequencing, showed that a somatic missense mutation (402C»G) occurred in four different AGCT samples at C134W (amino acid position 134) [15]. From the published results, the mutation exists in more than 97% of AGCT, and it is rarely detected in other ovarian cancer [16]. Some reports showed that the expression of FOXL2 was also downregulated in aggressive JGCT [17, 18]. These studies make FOXL2 as one possible pathognomonic defining feature. The mechanism of mutant FOXL2 in GCT was also widely explored. Some studies showed that prominent serine 33 (S33) phosphorylation of FOXL2, which is induced by GSK3β, was detected in C134W mutation. The phosphorylation modification of FOXL2 contributes to the growths of GCTs [19, 20]. The growth of GCT was proportional to the S33 phosphorylation status, and GSK3β inhibitor might serve as an effective intervention for GCT therapy [19].

Some studies examined the transcriptional targets of mutant FOXL2. Wile-type FOXL2 plays a key role in inhibiting granulosa cell proliferation and promoting apoptosis [21]. However, mutant FOXL2 downregulated the INHA, one of a proliferative signaling ligand [22]. Death signaling mediators, TNF-R1 (Tumor necrosis factor receptor 1) and FAS, were also decreased [23]. Caspase 8, BID and BAK determine the FOXL2 depended granulosa cell apoptotic pathway, but mutant FOXL2 was unable to elicit the apoptotic signaling responses [24]. In addition, mutant FOXL2 has been shown to reduce GnRH receptor expression, thus conferring resistance to GnRH-induced cell apoptosis [25]. Follistatin is mainly expressed in granulosa cells of developing follicles and it binds to activin A to block activin A-stimulated granulosa cells proliferation [26]. Mutant FOXL2 suppresses follistatin expression and leads to increased cells proliferation and tumor formation [6, 11]. Furthermore, FOXL2 mutation also leads to TGF-β signaling pathway deregulation [23].

More and more clinical data show that FOXL2 mutation is the main factor in AGCT So, understanding the FOXL2 regulation mechanism is instrumental to develop new prevention and therapy methods.

### Notch signaling pathway

Notch signaling is highly conserved in evolution, which plays critical role in organisms’ development. In mammalian, there are four Notch receptors, Notch1 to 4, There are three domains in Notch receptors, including functional extracellular (NECD), transmembrane (TM) and intracellular (NICD). Five Notch ligands, Jagged-1, Jagged-2, Delta-like-1 (DLL1), Delta-like-3 (DLL3) and Delta-like-4 (DLL4) had been identified [27, 28]. Both the Notch ligands and the receptors are transmembrane proteins. When ligands bind receptors, Notch receptors become susceptible to proteolytic cleavage mediated by secretase complex, which releases the intracellular domain of Notch. NICD enters nucleus and forms a complex with recombination signal binding protein-Jk, which contains a DNA binding domain. The complex regulates Myc, P21, HES family, Cyclin D3 and other Notch target genes. The deregulation of Notch signaling has been proved to be related to several cancers [27]. Studies showed that DLL4, Jagged-1, Notch1 and Notch4 were highly expressed in KGN cells (FOXL2-mutated granulosa tumor cell line), compared with granulosa-lutein cells. DAPT, an inhibitor of γ-secretase, was used to treat KGN cells and the inhibition of Notch system lead to lower proliferation and viability, as well as estradiol and progesterone secretion of KGN cells [13, 29]. Several apoptotic parameters such as BAX, BCLXs, PARP and caspase eight cleavages were increased after DAPT treatment. The interaction of Notch signaling and PI3K/AKT signaling were also been proved in the process. AKT phosphorylation was decreased and PTEN (phosphatase and tensin homolog deleted on chromosome ten) protein was increased after Notch signaling inhibition [13]. More studies are needed to demonstrate that Notch signaling would be potential therapeutic targets for AGCT.

### TGF-β signaling pathway

TGF-β super family is composed of 30 different growth and differential factors, including TGF-βs, activin, inhibin and BMPs (bone morphogenic proteins) [30]. TGF-β related signaling pathway plays critical roles in regulating stem cell cycle, organ development and immune cells through regulating cell proliferation, differentiation, and death [31]. SMAD proteins are general signaling pathway mediated proteins in TGFβ signaling network [32]. When the signaling pathway is abnormally activated, it may lead to diseases.

#### Activin and inhibin

Inhibin and activin are two hydrophilic non-steroid substances and are mainly synthesized by pituitary cells and ovarian granulosa cells. They have two α and β subunits. α and β subunits are encoded by different genes respectively. The β subunit consists of five different types of homologous compositions, βA, βB, βC, βD and βE. βA and βB units can form three kinds of activins linked by a disulfide bond, including activin A (βA-βA), B (βB-βB) and C(βA-βB) [33]. Inhibins were composed of Inhibin βA, βB units shared with activins, and a unique α-subunit (INHA), inhibin A (α-βA) and inhibin B (α-βB) [34]. Activins function via combining with activin-type 1 receptors and activin-type 2 receptors. The complex can activate SMAD2/3 through phosphorylation [35]. While inhibins carry out their biological role by antagonizing activin pathway [36].

Inhibin was first defined in 1932 [37]. As one gonadal hormone, Inhibin played important role in regulating folliculogenesis, steroidogenesis and FSH production [34]. Inhibin was also defined as a critical negative regulator of gonadal stromal cell proliferation and was identified to have tumour-suppressor activity [38]. Inhibin-α can inhibit granulosa cell proliferation and promotes apoptosis [39]. High expression of inhibin-α subunit exists in most human gonadal cancers. Loss of inhibin-α can lead to more aggressive GCT by using INHA knockout mice [38, 40–42]. As to the two subtypes of inhibin, studies showed that almost all the GCT patients had higher level of inhibin B, and over synthesized inhibin A was also detected in some cases [43]. Lower inhibin sensitive is also related to GCT development and tumor metastasis. Betaglycan or p120 was identified as inhibin receptors, which is associated with the type II or type I activin receptor subunits (ActR), respectively [44]. Studies showed that only p120 was especially high in GCT patients [44]. Not only in GCT, but also other types of ovarian tumor, abnormal elevated inhibin had been proved [43].

Activins have critical functions in genital system. Activin synthesized by pituitary gonadotrophic cells decreases the activation of luteotropic and promotes the expression of follicle stimulating hormone receptors, so it enhances the follicle development [45]. Activin binds to type II receptors, and then recruits and phosphorylates of type I receptor ALK4, which phosphorylates both SMAD2 and SMAD3 proteins. GCT cells show high inhibinβA subunit expression level and the proliferation rate was positively correlated with a high activin A to inhibin A ratio, suggesting that the tumor cells stimulated their growth through an activin A autocrine signaling pathway [46]. In GCT patients, abnormal activin receptors may lead to failure of granulosa cell apoptosis [26]. There are controversies about GCT markers. Early studies suggested that inhibin B and activin A were elevated in GCT patients. But recently it has also been suggested that inhibin B is more specific than activin A [47, 48]. In fact, activin still does not have the significance of large-scale application in the diagnosis and prognosis of GCT. The healthy activin levels are also increased significantly in postmenopausal women and ovarian cancer patients. The relationship between GCT and elevated inhibin is still unclear. The clinical application of inhibin as a tumor marker remains controversial. However, the clinical data and available studies suggest that the inhibin test may be used as part of a GCT screening and be used for disease prognosis [49, 50].

#### BMPs

Bone morphogenetic proteins (BMPs) are a group of at least 20 growth factors. They were firstly found in ostosis as induction proteins [51]. BMPs are multi-functional growth factors and have been implicated in a variety of functions. BMPs induce the formation of both cartilage and bone. BMPs also play a role in a number of non-osteogenic developmental processes, such as epidermal induction, inhibition of limb bud and myogenesis [52, 53]. BMPs can inhibit the growth of normal cells and human colon, prostate and breast cancer cell lines when the BMP signal components are complete. In addition, the BMP pathway was inactivated in 70% of colorectal cancers. The lack of two BMP type I receptors, Bmpr1a and Bmpr1b in the ovarian granulosa cells of mice were involved in the development of GCT [54]. The expression of Myc, cyclin D2 and cyclinE2 was highly increased in BMPR1a and BMPR1b double knockout mice, which result in the promotion of granulosa cell tumor proliferation [55]. BR-SMADs represented SMAD1/5/9 signal via interacting with BMP receptors. The study showed GCT development in BR-SMADs conditional knock out mice [56]. The studies in KGN cell line confirmed that FSH increased the expression of BMP type 1 receptors (BMPR1A/B) and BMP type 2 receptors (BMPR2). BMP6/7 induced phosphorylation of SMAD1/5/8. What’ more, FSH also promoted SMAD1/5 expression, while it reduced the inhibitory SMAD, SMAD6/7 expression [57].

As the upstream ligand for BMPR, BMP-2 is proved to be greatly associated with follicle formation. BMP7 was highly expressed in granulosa cell. Studies proved that BMP7 induced a rise rate in DNA synthesis and proliferation in granulosa cells [58, 59]. Studies showed that BMP15 was involved in polycystic ovary syndrome (PCOS). BMP15/SMAD1 may regulate granulosa cell apoptosis, which expression were significantly decreased in PCOS [60]. However, the relationship between BMPs and GCT is still unclear.

#### AMH

AMH is a regulator in sexual differentiation and it was also considered as an inhibitor in ovary cancer development. Normal granulosa cells and GCT both synthesize AMH. In human GCT, there was a controversy of high serum but low tissue AMH level. But it had been confirmed that serum AMH levels were positively correlate with the GCT size [61–63]. It can be speculated that AMH in the tumor microenvironment was lower than normal level [61]. AMH participates in the BMP signaling pathway to inhibit GCT formation, and lower expression of AMH reduced the activation of BMP signaling components. In addition, Extrinsic AMH reduced the number of KGN cells and primary GCT cells. It was proved that overexpression of AMH induced an increased activation of caspase-3 and subsequent apoptosis [64].

#### SMAD

SMADs are intracellular proteins, which occupy critical position in transducing TGF-β signaling from the receptors on cell surface to nucleus. According to the structures, SMADs divided in to three sub-families, R-SMAD (receptor-regulated type), Co-SMAD (common-mediator type) and I-SMAD (inhibitory type) [65]. In follicle development, many studies have showed these factors have co-operative functions.

SMAD 4 is the common mediator, is also a well-known tumor suppressor in human [66]. However, no ovarian tumors develop in SMAD4^loxp/loxp^ AMHR2 Cre mice. The deletion of SMAD4significantly disturbed the reproduction ability of mice. The lack of SMAD4 also promoted luteinization [67]. SMAD4 is necessary for FSH synthesis. Studies also indicated that SMAD4 played its role with FOXL2. When SMAD4 and FOXL2 were double deleted, FSH synthesis was almost stopped and females were sterile. The phenotype is similar to Fshb-knockout mice [68]. In ovulation period, SMAD4 was necessary in ERK1/2 activation [69]. During ovary development, the expression of SMAD4 was increased with the growth. SMAD4 silencing can increase the expression of CDK1 and CCNB2, suggesting that SMAD4 can also regulate cell cycle [70, 71].

Conditional deletion of SMAD1 and SMAD5 in ovarian granulose cells causes metastatic granulose cell tumors (GCTs) in female mice and phenocopies human juvenile GCTs (JGCTs) [54]. SMAD1/4/5 triple knockout mice showed increased survival rate and smaller tumor size, means that SMAD4 regulated signaling pathway may be a cancer promotor.

Both SMAD2 and SMAD3 are considered as important factors in ovarian development and functions. The ability of fecundity was reduced in the SMAD3-type (SMAD3-/-) mice, which also showed poor granulosa proliferation [72]. The TGF β-SMAD2/3 pathway is active in JGCTs. TGF-β may play as a promoting factor in JGCT, because the related signaling pathway can inhibit cell apoptosis [73]. Growing GCT cells expressed high levels of nuclear SMAD3 [46]. In KGN cell line, when NFκB expression is inhibited, the SMAD3 expression is also reduced, while SMAD3 is been proved overexpressed in AGCT [74]. SMAD3 interacted with FOXL2 and GATA4 modulated cell viability and apoptosis in ovarian granulosa cell tumour cells through regulating the expression of CCND2 [7, 75].

SMADs are critical regulation proteins in TGF-β family and they are significant in TGF-β signaling dynamic accommodation between cell plasma and nucleus. But the complete process is not clear now. The future study need to concentrate on the co-regulation among different SMADs and other regulating factors in GCT.

### PI3K/AKT signaling pathway

It has been demonstrated that activation of AKT pathway via PI3K is highly related to tumorigenesis. In ovarian cancers, AKT signaling blocks cell apoptosis through inhibiting FOXO1 and Bcl-2 proteins transcription [76, 77]. Forkhead box O1/O3, are the O class of the forkhead family. FOXO1 locates at chromosome 13, while FOXO3 locates at chromosome 6. In normal condition, FOXOs combine with the promotor of P27KIP1. P27 (Cyclin-dependent kinase inhibitor 1B) is an inhibitor in cell cycle. Once FOXOs were phosphorylated by AKT, the transcription of p27 would be influenced and lead to abnormal cell proliferation. [78]. Evidences showed FOXO1a influenced p27 nuclear localization and inhibited granulosa cell proliferation [79]. TRAIL has the effect of high efficiency and rapid induction of apoptosis of tumor cells and virus infected cells, but not normal cells [80]. FOXOs can also regulate TRAIL [81]. The knockdown of TRAIL obviously reduced the apoptosis induced by FOXO3 [82]. FOXO1/3 double knock out mice have high risk in getting GCT [83].

PTEN is one negative regulator of PI3K signaling and its mutation is very common in human cancers. PTEN is an important tumour suppresser. Studies indicated that lower PTEN expression levels was related to cancer development [84]. The expression of PTEN in ovarian cancer was negatively correlated with clinical stage, differentiation and VEGF expression. The overall survival of PTEN-positive patients was significantly longer than that of negative expression [85]. The depletion of PTEN and FOXO1/3 has a synergistic effect in GCT development, but the single knockout PTEN mice rarely developed to GCT [83]. In ovaries, PTEN mutation leaded to over-phosphorylation of AKT. In addition, FOXOs can promote PTEN transcription, suggesting that there exists a negative feedback among them [81, 86]. In addition, uncontrolled PI3K activity within oocytes irreversibly transforms granulosa cells into GC tumors through perturbed local cell communication [46]. Lague et al. provided powerful evidence for the role of the dysregulation of the PI3K/AKT pathway in the pathogenesis of GCTs by means of a Pten^flox/flox^, Amhr^cre/+^ mouse model. Activation of WNT/CTNNB1 signaling causes late-onset GCT development. However, activation of both the PI3K/AKT and WNT/CTNNB1 pathways in the granulosa cell. Had 100% penetrance and extremely rapid growth and the ability to spread [87].

### Others

#### GATA4

GATA4 belongs to the GATA family of zinc finger transcription factors. In human, it locates at 8p23.1. The current study indicated that GATA4 played key role in embryogenesis and cardiac development. GATA4 mutations can lead to heart disease due to abnormal fold or other embryogenic failure in heart. Based on statistics of clinical GCT samples, overexpression of GATA-4 is associated with higher recurrence and more aggressive of GCT [63]. Evidences show GATA4 can regulate GCT cell apoptosis and proliferation by activating Bcl-2 and cyclin D2, respectively [88, 89]. Overexpressing GATA4 protects GCTs from TRAIL-induced apoptosis in KGN cells [90]. In other species, like dogs, GATA-4 is also the GCT markers [91]. In previous sections, we introduced the role of SMAD3 and FOXOL2. Studies showed that FOXL2, GATA4, and SMAD3 expression patterns overlap in the fetal and adult ovary. The three factors co-operatively modulate the cell viability and apoptosis in ovarian granulosa cell tumour cells through regulating the expression of CCND2 [7].

#### VEGF

Vascular endothelial growth factor played important role in cancer development, as a cell factor that mainly regulates vasculogenesis. VEGF and its receptors were hugely expressed in GCTs [92, 93]. In anti-VEGF therapy, some case reports showed that bevacizumab was benefited to recurrent GCT patients [93]. VEGFR1 normally be considered as a decoy receptor and it showed unobvious phosphorylation when combined with VEGF [94]. VEGFR2, was usually seen as the main mediator of VEGF [95]. The expression levels of VEGFR2 were higher in GCT patients, while VEGF1 expression levels showed no increase trendy [93].

## Conclusion

**Fig. 1:**
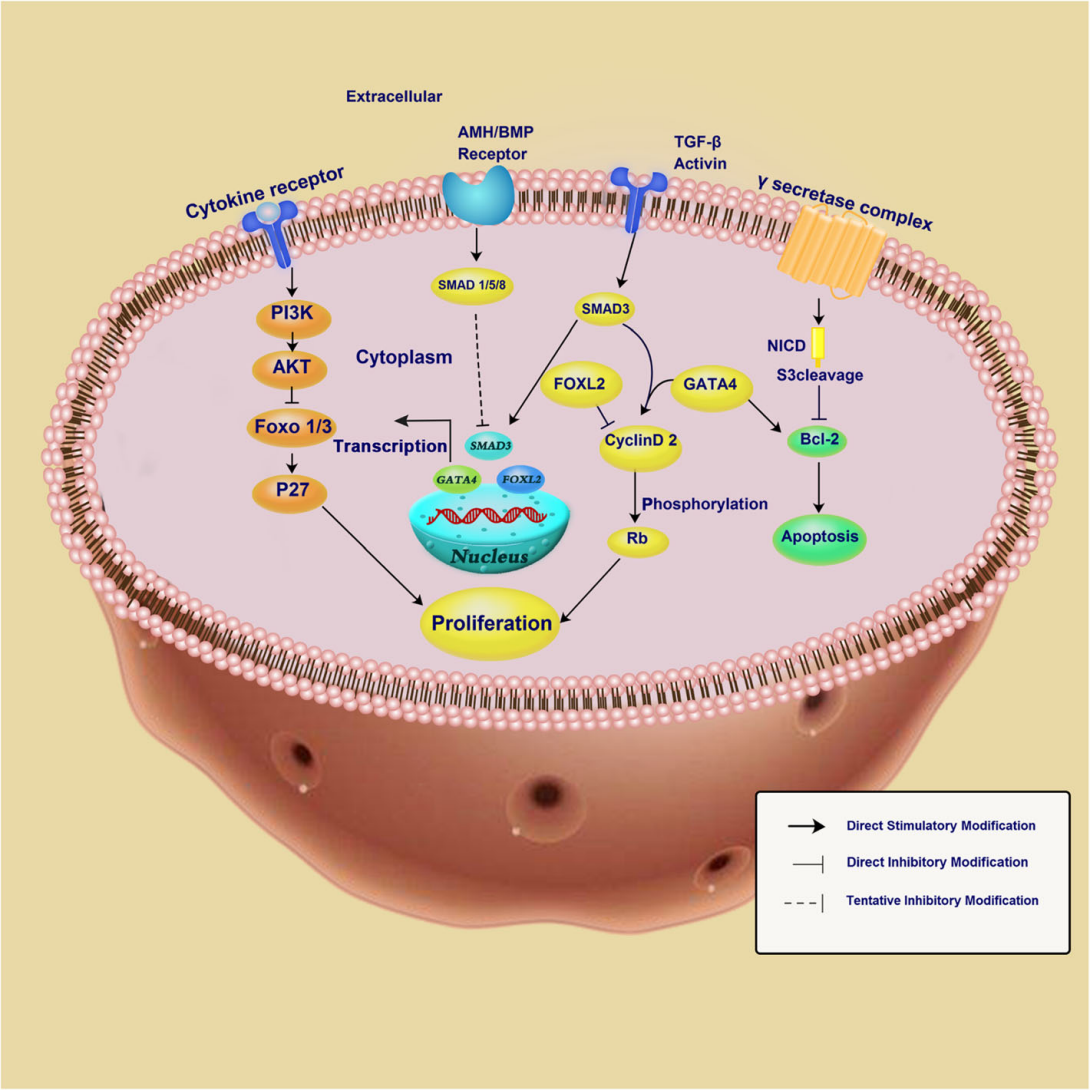
Schematic representation of the cell signaling pathways in GCT development. PI3K, phosphatidylinositol-3-kinase; AKT, serine/threonine kinase; FOXO 1/3, forkhead box O1/3; AMH, anti-Mullerian hormone; BMP, bone morphogenetic protein; SMAD, drosophila mothers against decapentaplegic protein; FOXL2, forkhead transcription factor 2; Rb, retinoblastoma protein; Bcl-2, B-cell lymphoma 2

GCT incidence of ovarian cancer accounts for about 2% to 5%, while the prognosis is usually better. Five-year survival rate is up to 90%. High recurrent rate is the most critical factor for GCT death. At present, the most important problem lies in the early diagnosis and prevention of recurrence. FOXL2 mutation is the main cause of GCT, while other factors also contributed to it, such as GATA4, SMAD, VEGF, PI3K/AKT, AMH and TGF-β.The \treatment for GCT includes surgery, radiotherapy, chemotherapy and hormone therapy. Studies showed that hormones play a critical role in the pathogenesis and treatment of GCT, especially in some ineffective cases for radiotherapy and chemotherapy. However, the serious adverse effects were followed. New treatment strategies need to be explored. The employment of anti-VEGFA antibody in mouse model successfully slowed the tumor development through inhibiting the tumor cell proliferation [96]. Other studies also demonstrated anti-VEGF (or VEGFR) had conspicuous function in inhibiting GCT development [97, 98]. As for chemotherapy, drug targeting in critical signaling pathways can increase efficiency. Chemokine (C-C motif) ligand 5 (CCL5) can reduce the toxic of cisplatin by increasing AKT phosphorylation level [99, 100]. GLS (glutaminase) is the key metabolic enzymes of glutaminolysis, which is highly expression in tumors. The inhibition of GLS1 leaded to decreased expression of phosphorylated STAT3, which provided an idea in overcoming the resistance of PI3K inhibitor [100, 101]. Targeted therapy for signaling pathways can effectively prevent the recurrence of GCT and reduce the resistance of chemotherapeutic drugs. Many studies have shown that GCT-related signaling pathways and pathogenic genes are closely linked, and the link between these signaling pathways is unclear. As shown in Fig. 1, we summarized these related signaling pathways network. Certainly, surgery is still the most important treatment. Targeting drugs for signal pathway in the subsequent chemotherapy can significantly improve the survival rate of patients. Future research should further focus on exploring the molecular mechanisms of GCT and developing targeted medicines. Prevention of recurrence of GCT will significantly improve patient survival.

## Abbreviations

AGCT: Adult granulosa cell tumor
AKT: Serine/threonine kinase
AMH: Anti-Mullerian hormone
Bcl-2: B-cell lymphoma 2
BMP: Bone morphogenetic protein
DLL1: Delta-like-1
DLL3: Delta-like-3
DLL4: Delta-like-4
FOXL2: Forkhead box L2
FOXO 1/3: Forkhead box O1/3
GCT: Granulosa cell tumor
GLS: Glutaminase
INHA: Inhibin α-subunit
JGCT: Juvenile granulosa cell tumor
PI3K: Phosphatidylinositol-3-kinase
PTEN: Phosphatase and tensin homolog deleted on chromosome ten
Rb: Retinoblastoma protein
SMAD: Drosophila mothers against decapentaplegic protein
TGF-β: Transforming growth factor beta
VEGF: Vascular endothelial growth factor
